# Smartphone Accelerometer Assessment of HRV: Concurrent Agreement of the KubiosHRV App

**DOI:** 10.3390/s26216837

**Published:** 2026-10-29

**Authors:** Alfonso Cervantes-Mugüerza, Alejandro Martínez-Cava, Jesús G. Pallares, Elena Conesa-Ros, Jesús J. Ruiz-Navarro

**Affiliations:** Human Performance and Sports Science Laboratory, Faculty of Sport Sciences, University of Murcia, 30720 Murcia, Spain; alfonso.c.m@um.es (A.C.-M.); alejandro.martinez12@um.es (A.M.-C.); jgpallares@um.es (J.G.P.); econesaros@um.es (E.C.-R.)

**Keywords:** HRV, autonomic modulation, monitoring, athletes, application

## Abstract

Heart rate variability (HRV) is widely used to monitor athlete recovery and adaptation. Recent technological advances have enabled HRV assessment through smartphone applications without the need for external devices. This study aimed to assess the concurrent agreement between the accelerometer-based HRV measurement feature of the KubiosHRV app and the Polar H10 in supine, seated, and standing positions. A total of 52 participants (30 males and 22 females) across performance tiers 0–4 completed a 2 min resting recording, with the first minute discarded and the second minute analysed in each position. HRV was measured simultaneously using a Polar H10 chest strap and the accelerometer-based feature of the KubiosHRV app on an iPhone 14. The analysed variables included Mean RR, RMSSD, SDNN, LF power, HF power, SD1, and SD2. Data were automatically processed by the KubiosHRV app and manually extracted into a database. Results showed excellent intraclass correlation coefficients (≥0.985) and small magnitudes of error for time-domains and non-linear variables in the three analysed positions with the reference criterion. Frequency-domain variables showed larger error estimates. Bland–Altman analysis showed no evidence of proportional bias across the measurement range (*p* > 0.05). These findings suggest that, during 1 min recordings, the accelerometer-based feature of the KubiosHRV app showed strong concurrent agreement with the Polar H10 for time-domain variables and acceptable agreement for selected non-linear variables across positions. Therefore, it may represent a practical alternative for routine monitoring of these HRV metrics when external hardware is unavailable.

## 1. Introduction

Heart rate variability (HRV) is a valid physiological tool for monitoring the activity of the autonomic nervous system [1,2], which has been explored in response to various factors, such as training or psychological stress [3,4]. In this regard, HRV is monitored to provide insight into athletes’ adaptations to training during specific periods of the competitive season [1,5,6]. Understanding how an athlete’s autonomic nervous system responds to training stress can aid coaches and support staff adjusting future training loads, facilitating improved recovery and enhancing performance potential [5,6,7].

Due to its ease of use and non-invasive characteristics, HRV has gained popularity over the recent years in elite athletes, clinical patients, and the general population [8,9,10]. This broader accessibility to the general population is largely attributable to the development of different devices that facilitate the measurements, ranging from early electrocardiograms to heart rate monitor sensors (e.g., chest straps) and, more recently, photoplethysmography-based devices [11,12,13]. In this regard, a new feature based on smartphones’ accelerometers has recently been developed to assess HRV [14]. More specifically, the KubiosHRV app has introduced an accelerometer-based HRV measurement feature that is designed to test HRV without the need of any external device and to be easily incorporated into daily routine. This may be particularly valuable for routine assessments, which are often limited by logistical challenges, device management, user compliance, and associated costs.

By utilising the accelerometer integrated into smartphones, the app is able to derive various HRV metrics. Based on the principles of ballistocardiography and seismocardiography, small mechanical forces generated by cardiac activity and transmitted through the body are detected by the accelerometer. Then, advanced signal processing algorithms convert these subtle vibrations into interbeat interval data (the same type of data used for calculating HRV). Finally, from this metric, HRV parameters are provided by the app [14]. However, to the best of our knowledge, the concurrent agreement between HRV measurements derived from its accelerometer-based feature and those obtained using a Polar H10 chest strap has not yet been evaluated. Therefore, this study aimed to assess the concurrent agreement between the accelerometer-based HRV measurement feature of the KubiosHRV app and the Polar H10 in supine, seated, and standing positions. Specifically, this study contributes by: (i) evaluating agreement between smartphone accelerometer-derived and Polar H10-derived HRV measurements obtained within the same app environment; (ii) examining agreement across three commonly used body positions; and (iii) providing metric-specific estimates of relative and absolute error, including Bland–Altman analysis, for 1 min HRV recordings.

## 2. Materials and Methods

### 2.1. Design

A cross-sectional study design was used to evaluate concurrent agreement between HRV measurements derived from the accelerometer-based feature of the KubiosHRV mobile application (version: 1.7.13, Kubios Oy, Kuopio, Finland) and those derived from a Polar H10 chest strap (Polar Electro Oy Inc., Kempele, Finland), both processed using the same application. The Polar H10 served as the criterion standard as it is currently considered an industry standard for HRV data collection, having been previously validated against ECG-based HRV measurement methods [13]. Data were collected using two iPhone 14 devices (iOS 26, Apple Inc., Cupertino, CA, USA): one using the accelerometer-based HRV measurement feature and the other connected to the H10 chest strap. Data collection occurred in a single session for each participant under standard conditions (20–24 °C air temperature; 45–55% humidity). Participants completed a single 2 min recording in each of the supine, seated, and standing positions (same order for all participants) while simultaneously wearing both the accelerometer and the H10 to enable comparison of HRV across devices. A 5 min interval was provided between recordings in the different postural conditions.

### 2.2. Participants

A heterogeneous sample of 52 participants (30 males and 22 females) volunteered to take part in this study. According to the performance classification proposed by McKay et al. [15], participants were categorised into performance tiers ranging from 0 to 4 (see Table 1). A diverse sample was collected to capture a wide spectrum of HRV values (low–high) across different types of athletes, from sedentary individuals (i.e., tier 0) to athletes competing at the international level (i.e., tier 4), encompassing physical states ranging from fatigued to fully recovered. The protocol, benefits, and risks associated with participation in the study were explained to each subject or their parents (i.e., participants under 18 years old) before providing consent to participate. The study was conducted according to the code of ethics of the World Medical Association (Declaration of Helsinki), and the protocol was approved by the University of Murcia Ethics Committee (M10/2025/544).

### 2.3. Procedures

Anthropometric measurements were collected from each participant during the week before the testing day by an International Society for the Advancement of Kinanthropometry Level 1-accredited researcher. Height and body mass were evaluated using a stadiometer and body scale (Seca 799, Hamburg, Germany). The body mass index was computed as follows: body mass (kg)/height (m)^2^.

On the testing day, participants arrived at the facilities and were fitted with the H10 chest strap, which was connected to the KubiosHRV app on an iPhone 14. Moreover, a second iPhone 14 using the accelerometer function was placed flat on the participants’ chest for supine measurements. To prevent the phone from falling during the seated and standing conditions, the iPhone 14 was secured with a strap in these conditions. A standardised manual synchronisation procedure was applied for each trial. A single researcher delivered a verbal countdown and triggered both iPhones concurrently at its completion to obtain paired recordings. To enhance the reliability of the measurements all the procedures were always performed by the same researcher.

Among the two available features in the KubiosHRV app, both devices were configured to use the “Readiness Measurement” mode. All recordings were analysed within the app using the same standardised automated workflow and identical in-app settings for both devices. No manual editing of R–R/interbeat interval data, artefact correction, or post-processing was performed by the researchers. Moreover, in the “Readiness Measurement” mode, the app provides a qualitative signal-quality rating of “very low”, “low”, or “good”. Only recordings classified as “good” were retained for analysis; when a recording received a lower rating, it was excluded, and the participant was reassessed using both devices together following the same standardised procedure. Of the 156 measurements (52 participants × 3 measurements), 6 accelerometer-based measurements (2 supine, 4 standing), which represents 3.8% of measurements, did not meet the app-defined signal-quality criterion of “good”; in each case, measurements from both devices were repeated simultaneously again. Consequently, all paired observations included in the analysis met the app-defined signal-quality criterion and were processed under identical analytical conditions. Accordingly, the entire processing workflow was limited to the app’s native functions to reflect its intended real-world use and maximise ecological validity.

Participants were instructed to remain quiet, to keep both eyes open, to refrain from falling asleep, to try not to move the body, and to breathe spontaneously during the test [16]. More specifically, in the supine position, participants lay flat on their backs with their arms resting at their sides. In the seated position, each participant was instructed to sit on a chair without armrests, keeping their feet flat on the floor [17]. Finally, in the standing position, participants remained upright and still, with their arms relaxed at their sides. Within the single 2 min recording per postural condition, the first minute was used for stabilisation and discarded and the second minute was used for HRV analysis [10,16,18,19]. Participants were tested in the following order: supine, seated, and standing in a quiet indoor facility. The following parameters, which are categorised as time-domain measurements, were automatically processed by KubiosHRV app and manually extracted into a database: Mean RR, the mean of the time interval between 2 consecutive R waves or intervals in case of the accelerometer; RMSSD, squared root of the mean of the sum of the squares of successive normal R–R interval differences; and SDNN, standard deviation of all normal R–R intervals. These variables were selected from those provided by the KubiosHRV app as they are the most robust indicators for short-duration HRV recordings and are commonly used in athletic populations [20,21]. For the accelerometer-based method, the aforementioned HRV metrics were derived from interbeat intervals estimated from the mechanical cardiac signal rather than from ECG-derived R–R intervals. Moreover, as the KubiosHRV app also provides frequency-domain and non-linear indices, LF (absolute power in the low-frequency band, 0.04–0.15 Hz), HF (absolute power in the high-frequency band, 0.15–0.40 Hz), SD1 (Poincaré plot standard deviation perpendicular to the line of identity), and SD2 (Poincaré plot standard deviation along the line of identity) were additionally extracted and entered into the database.

Factors such as the time of measurement, prior food intake, or recent physical activity might influence HRV values; however, given that the primary aim of the study was to assess the concurrent agreement between the two measurement methods, these variables were not controlled. In fact, the inclusion of participants with a wide range of HRV values enhanced the robustness of the sample by allowing comparison across a broader spectrum of physiological states.

### 2.4. Statistical Analysis

For the analysis of concurrent agreement, both absolute and relative levels of agreement, as well as the magnitude of measurement error, were assessed. Intraclass correlation coefficients (ICC) (A,1; two-way mixed-effects, absolute agreement, single measurement model) were calculated following the guidelines of Koo and Li [22], together with their 95% confidence intervals. ICC values were interpreted as follows: <0.50, poor; 0.50–0.75, moderate; 0.75–0.90, good; and >0.90, excellent [22]. The width of the 95% confidence intervals (95% CI) was used to assess the precision of the ICC estimates. The standard error of measurement (SEM) was obtained from the square root of the mean square error term in a repeated-measures analysis of variance [23]. Results were reported both in absolute and relative terms using the coefficient of variation (CV = 100 × SEM/mean). For most sport performance tests and sport technologies, CV values are recommended to be below 5% [24,25]. Additionally, the smallest detectable change (SDC) was calculated as √2 × SEM × 1.96 [26] and interpreted as the minimum between-method difference required to exceed the measurement error observed in the concurrent comparison. It was not interpreted as a threshold for longitudinal change or between-day reliability.

Bland–Altman plots were generated to assess agreement between devices. The bias (mean difference: accelerometery − PolarH10), its 95% CI, and upper and lower limits of agreement (defined as mean difference ± 1.96·SD), with the corresponding 95% CI, were also calculated [27]. For each HRV variable and body position, proportional bias was assessed using linear regression of the between-method differences on the mean of the two methods. A regression slope significantly different from zero was considered evidence of proportional bias. Heteroscedasticity was subsequently evaluated from the residuals of this regression using the Breusch–Pagan test. The White test, including both the mean measurement magnitude and its squared term, was used as a complementary assessment of non-linear changes in residual variance. Statistical significance was set at *p* < 0.05. Spearman correlations between the absolute residuals and measurement magnitude, influence diagnostics, leave-one-out analyses, and analyses after excluding residuals with absolute studentised values greater than 3 were used to assess the robustness of the findings. The analyses were also repeated on the logarithmic scale to determine whether this transformation stabilised the residual variance.

ICC, SEM, CV, SDC, and Bland–Altman statistics were calculated using SPSS version 25.0 (IBM Corp., Armonk, NY, USA), and the Bland–Altman plots were generated using GraphPad Prism version 6.0 (GraphPad Software Inc., Boston, MA, USA). Heteroscedasticity, proportional bias, influence, and sensitivity analyses were conducted using R version 4.5.2 (R Foundation for Statistical Computing, Vienna, Austria).

## 3. Results

**Figure 1:**
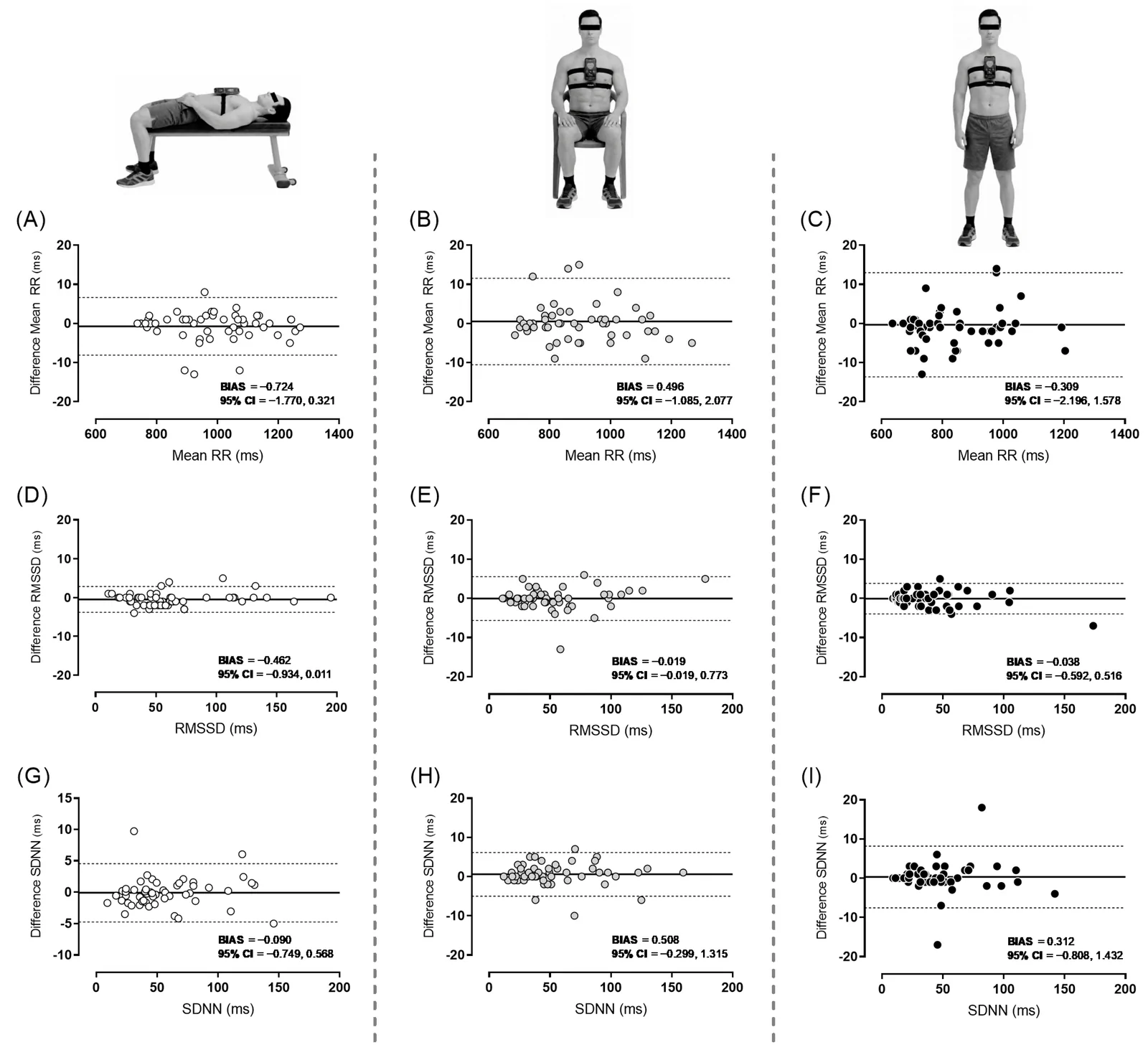
Bland–Altman plots of the H10 and accelerometer devices for time-domain heart rate variability measurements, with bias (solid black line) and lower and upper limits of agreement (dashed lines). (**A**) H10-measured Mean RR and accelerometer-measured Mean RR in supine position; (**B**) H10-measured Mean RR and accelerometer-measured Mean RR in seated position; (**C**) H10-measured Mean RR and accelerometer-measured Mean RR in standing position; (**D**) H10-measured RMSSD and accelerometer-measured RMSSD in supine position; (**E**) H10-measured RMSSD and accelerometer-measured RMSSD in seated position; (**F**) H10-measured RMSSD and accelerometer-measured RMSSD in standing position; (**G**) H10-measured SDNN and accelerometer-measured SDNN in supine position; (**H**) H10-measured SDNN and accelerometer-measured SDNN in seated position; (**I**) H10-measured SDNN and accelerometer-measured SDNN in standing position.

**Figure 2:**
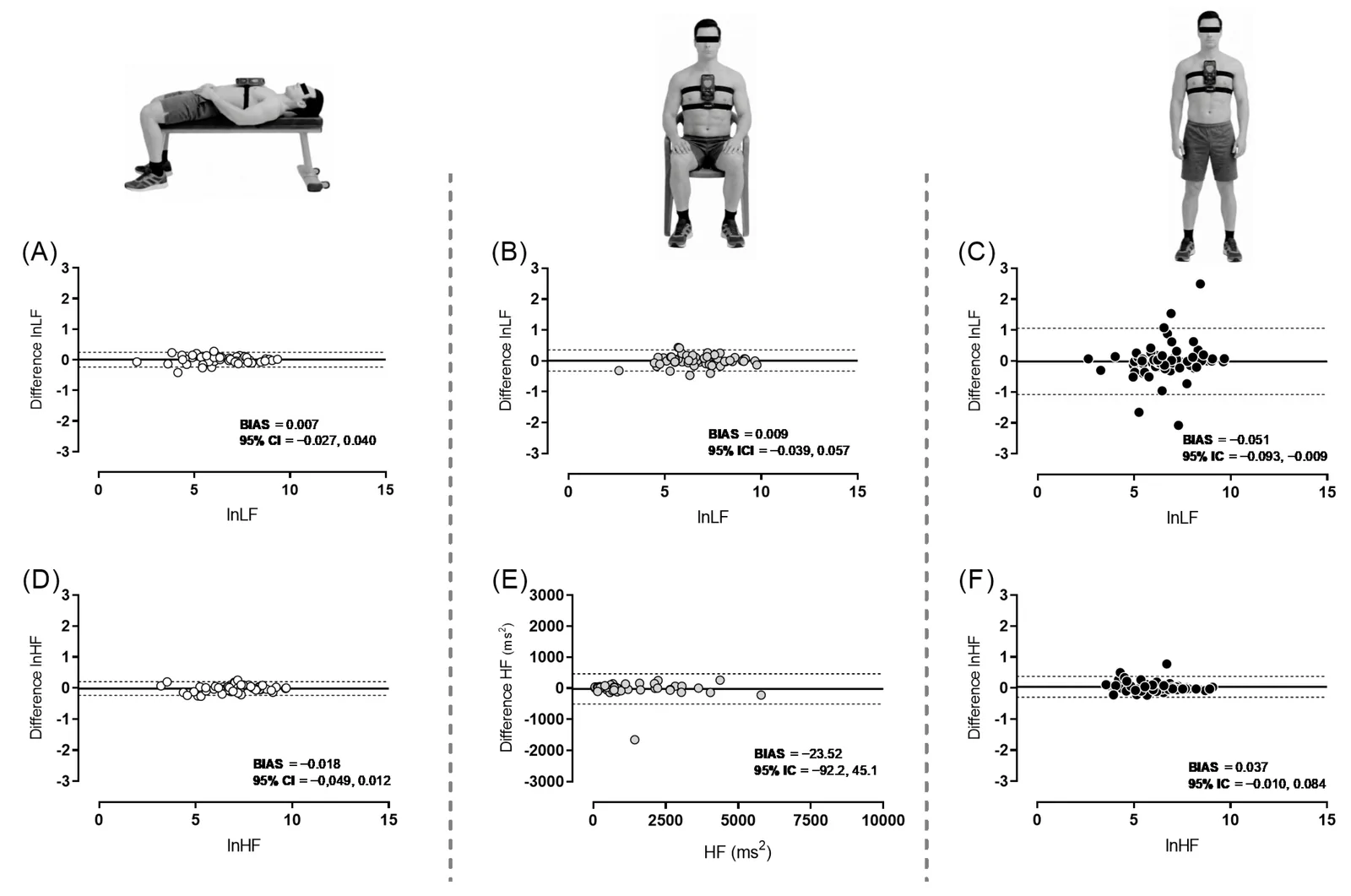
Bland–Altman plots of the H10 and accelerometer devices for frequency-domain heart rate variability measurements, with bias (solid black line) and lower and upper limits of agreement (dashed lines). (**A**) Natural-log-transformed H10-measured and accelerometer-measured LF in supine position; (**B**) natural-log-transformed H10-measured and accelerometer-measured LF in seated position; (**C**) natural-log-transformed H10-measured and accelerometer-measured LF in standing position; (**D**) natural-log-transformed H10-measured and accelerometer-measured HF in supine position; (**E**) H10-measured and accelerometer-measured HF in seated position; (**F**) natural-log-transformed H10-measured and accelerometer-measured HF in standing position. ln: natural logarithm.

**Figure 3:**
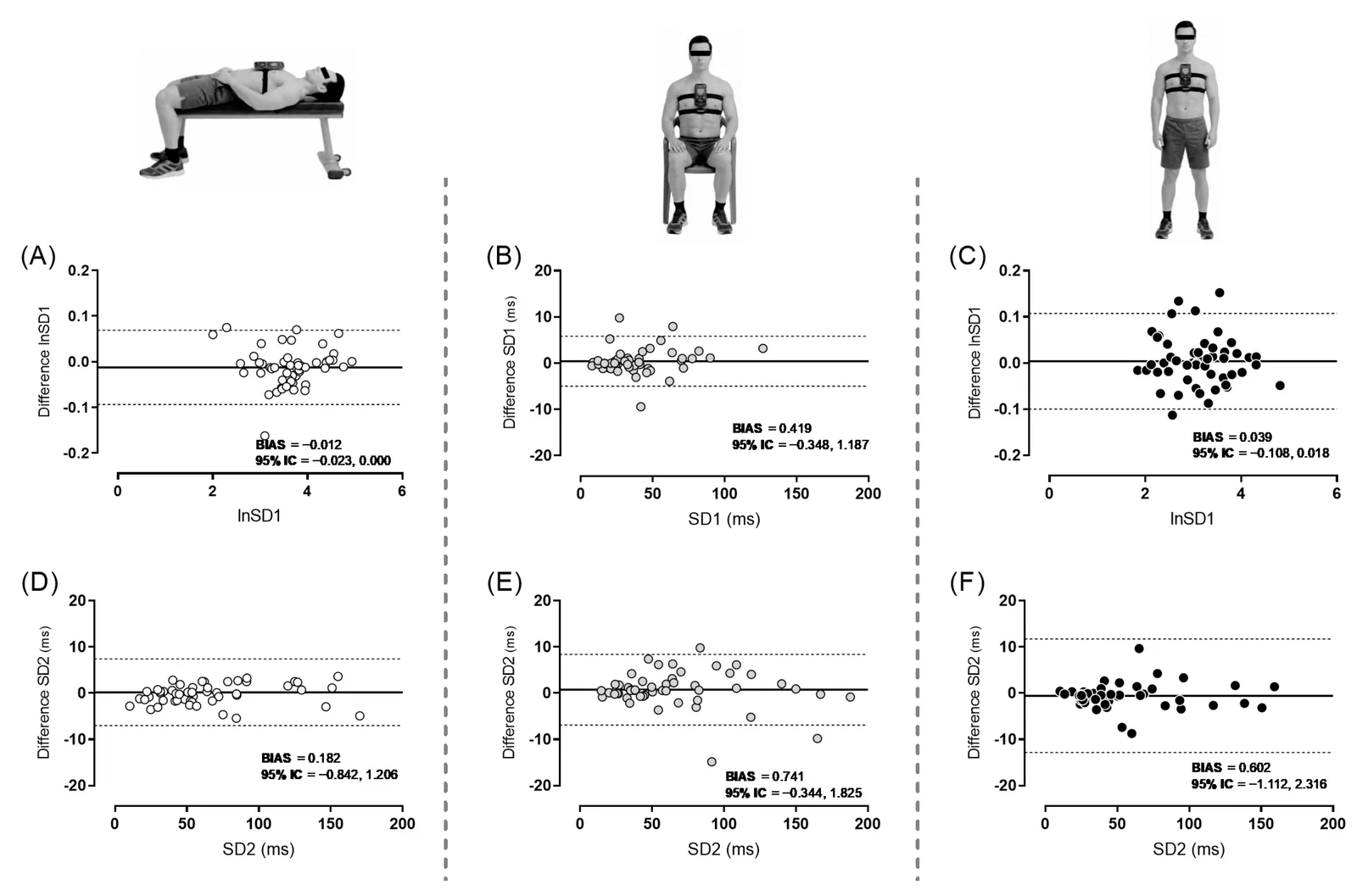
Bland–Altman plots of the H10 and accelerometer devices for non-linear heart rate variability measurements, with bias (solid black line) and lower and upper limits of agreement (dashed lines). (**A**) Natural-log-transformed H10-measured SD1 and accelerometer-measured SD1 in supine position; (**B**) H10-measured SD1 and accelerometer-measured SD1 in seated position; (**C**) natural-log-transformed H10-measured SD1 and accelerometer-measured SD1 in standing position; (**D**) H10-measured SD2 and accelerometer-measured SD2 in supine position; (**E**) H10-measured SD2 and accelerometer-measured SD2 in seated position; (**F**) H10-measured SD2 and accelerometer-measured SD2 in standing position. ln: natural logarithm.

Table 2 presents the ICC, 95% limits of agreement, SEM, CV, and SDC for the accelerometer and H10 devices for Mean RR, RMSSD, SDNN, LF, HF, SD1, and SD2 in the supine, seated, and standing positions. Overall results showed excellent agreement (≥0.985) in all the variables measured. The lower bound of every 95% CI was ≥0.974, indicating consistently precise ICC estimates. Very small magnitudes of error (limits of agreement, SEM, CV, and SDC) were observed for time-domain and slightly greater errors in non-linear variables. In contrast, frequency-domain variables showed larger error estimates, with CVs exceeding 5%. The Bland–Altman plots for time-domain, frequency-domain and non-linear HRV variables across the three assessed positions are shown in Figure 1, Figure 2 and Figure 3, respectively. Apart from the small bias, Bland–Altman analysis showed no proportional bias across the measurement range (*p* > 0.05). Evidence of heteroscedasticity was observed for LF in all three positions and for HF and SD1 in the supine and standing positions. Therefore, Bland–Altman analyses for these comparisons were performed on natural-log-transformed data, and the limits of agreement were back-transformed and reported as ratios. However, after natural logarithmic transformation, residual evidence of heteroscedasticity remained for supine LF, supine HF, and standing LF. Therefore, the corresponding limits of agreement should be interpreted with caution, as constant limits may not represent agreement uniformly across the full measurement range.

## 4. Discussion

This study aimed to assess the concurrent agreement between the accelerometer-based HRV measurement feature of the KubiosHRV app and the Polar H10 in supine, seated, and standing positions. Overall, the results showed excellent ICC, with narrow 95% CI, for all the variables analysed across the three measured positions relative to the reference criterion (H10 chest strap). Moreover, it showed very small magnitudes of error (Bias, Limits of agreement, SEM, CV, and SDC) for time-domain and slightly higher error for non-linear variables. In contrast, frequency-domain variables showed larger error estimates, with CVs exceeding the 5% threshold. These findings suggest that the accelerometer-based HRV measurement feature of the KubiosHRV app may be a practical alternative to the Polar H10 for assessing non-linear and, specially, time-domain HRV variables from 1 min recordings in supine, seated, and standing positions.

HRV results were slightly stronger in the supine position compared with the seated and standing positions. During the seated and standing conditions, the smartphone had to be secured with a strap to prevent displacement, which may have introduced minor additional movement or variability in the accelerometer-derived signal, potentially contributing to the slightly lower agreement observed in these positions [14]. In this regard, it is currently difficult to establish what constitutes an acceptable bias based on the existing literature, as differences in experimental settings and analytical methods substantially influence these values [28]. However, considering for instance that RMSSD values range in this study from ~25 to 200 ms, the bias reported (<0.5 ms) can be regarded as negligible relative to the physiological range observed [11]. Indeed, the mean biases observed in the present study were substantially smaller in magnitude than those reported in previous validation studies. For instance, O’Grady et al. [29] found that the Apple Watch underestimated SDNN by 8.31 ms relative to the Polar H10 during supine rest, compared with a mean bias of 0.09 ms in the present study. Similarly, Stone et al. [17] reported mean biases of −0.74 ms for RMSSD obtained using HRV4Training with an ECG chest strap and −2.33 ms for Oura, both compared with multilead ECG during seated rest, whereas the present study showed a mean bias of −0.019 ms. However, these studies used 5 min recordings, whereas the present study used a 1 min protocol. Direct comparisons should be avoided as the studies differed in recording duration and population, as well as, in the case of Stone et al. [17], the reference method.

Furthermore, while no consensus thresholds currently exist for interpreting the practical magnitude of limits of agreement in HRV validation research, the limits observed in the present study were comparable to those reported in previous validation studies [12]. In addition, other methodological considerations such as food or coffee intake, sleep duration and schedule or room conditions may induce greater changes in HRV than the biases reported in this study [30]. Thus, based on the present findings, non-linear and, particularly, time-domain variables can be monitored using the KubiosHRV app with a likely small bias, without the need for additional hardware. This may be particularly valuable for routine assessments in applied settings, or as a substitute when devices such as the H10 are not available, with reasonable confidence that the derived metrics will be comparable.

In contrast, frequency-domain variables showed the weakest agreement and the largest error estimates, with CVs exceeding the prespecified 5% threshold [24,25]. Frequency-domain estimates are highly sensitive to recording duration, and the 1 min protocol used in the present study was likely insufficient [31]. For instance, Shaffer et al. [32] recommended a minimum duration of 90 s for LF and 180 s for HF. Recent evidence has also indicated that the reliability of both components improves as recording duration increases [33]. Moreover, frequency-domain comparisons exhibiting heteroscedasticity showed increasing residual dispersion at higher spectral-power values, indicating greater absolute between-device variability as measurement magnitude increased. Accordingly, the frequency-domain findings of this study should be considered exploratory. Future studies should evaluate their agreement using longer recording windows, such as the standard 5 min recording period, or shorter durations specifically validated for each spectral component.

Although the app was initially designed for HRV assessment in the supine position, probably because the highest global HRV is usually measured in that position [34], the present study sought to evaluate concurrent agreement between the two measurement methods in the seated and standing positions as well, given the known influence of body position on HRV responses and the additional insights that these postures can provide [30,34]. More specifically, the standing position reveals additional insights into cardiac autonomic adaptability and training status that supine measurements may miss [35]. Therefore, athletes and coaches may measure HRV in a different position than supine or conduct an orthostatic test involving transitioning between multiple positions [34]. Hence, despite the use of a strap to secure the device, assessing agreement across the different positions was necessary to ensure a more comprehensive evaluation and to enhance its applicability to a broader range of users.

Despite the overall favourable results observed across all analysed variables, the strongest agreement was found for the time-domain measures. These could be expected as these variables are the most robust indicators for short-duration HRV recordings and are commonly used in athletic populations [20,21]. Importantly, our primary aim was not to validate the physiological interpretation of these metrics but rather to evaluate the performance of the device itself. Therefore, the interpretation of the physiological meaning of the analysed variables should be approached with caution.

It is important to acknowledge that this study focused on short recording durations and, therefore, the observed agreement may not necessarily extend to longer measurement periods. Future research should examine these metrics under longer recording conditions to further establish their robustness. Moreover, recordings were conducted using a single smartphone model. Although the KubiosHRV app remains consistent across devices, hardware differences between smartphone models may influence performance and, thus, potential variability across devices should be considered. Furthermore, although a standardised manual synchronisation procedure was used, the two iPhones were not synchronised through a shared hardware or software trigger. Therefore, a small temporal offset between the recording start times cannot be excluded, which may have influenced the agreement estimates obtained from these ultra-short HRV recordings. Future studies should use a common trigger or automated synchronisation procedure to ensure precise temporal alignment between devices. Finally, both signals were processed within the KubiosHRV app, resulting in a partially shared analytical workflow that may have influenced the observed agreement. However, this approach was intentional, as applying the same processing protocol to both signals helped ensure that any observed differences were primarily attributable to the measurement method rather than to post-processing procedures. Future studies should compare simultaneously acquired accelerometer-, chest-strap-, and ECG-derived interbeat/R–R intervals and HRV metrics using independent processing pipelines.

## 5. Conclusions

The accelerometer-based HRV measurement feature of the KubiosHRV app demonstrated metric-dependent concurrent agreement with the Polar H10 during 1 min recordings performed in supine, seated, and standing positions. Time-domain variables showed the strongest agreement and the smallest errors, whereas non-linear variables exhibited slightly greater errors. In contrast, frequency-domain variables showed weaker agreement and CVs exceeding the prespecified 5% threshold. These findings suggest that, when recordings meet the app-defined “good” signal-quality criterion, the app can provide a practical alternative to the Polar H10 for short resting assessments of time-domain and selected non-linear HRV variables. However, frequency-domain estimates obtained from 1 min recordings should be considered exploratory and should not be regarded as interchangeable with those derived from the Polar H10. Although a strap is required to secure the smartphone during seated and standing measurements, the approach does not require the need for additional external hardware. This simplified approach may facilitate the routine assessment of HRV in a wide range of settings, particularly among recreational athletes, individuals with limited access to monitoring equipment, and practitioners seeking a practical solution for regular HRV monitoring. Future studies should evaluate frequency-domain agreement using standard 5 min recordings, compare the method with simultaneous criterion ECG, determine its between-day reliability, and assess its performance in broader populations and less controlled conditions.

## Figures and Tables

**Table 1:** Participants’ main characteristics presented as mean ± standard deviation [range].

	Tier 0 (*n* = 10)	Tier 1 (*n* = 13)	Tier 2 (*n* = 13)	Tier 3 (*n* = 10)	Tier 4 (*n* = 6)
Age (years)	32.7 ± 15.8 [19, 63]	22.6 ± 4.1 [19, 33]	23.1 ± 5.5 [20, 40]	24.3 ± 9.5 [17, 50]	25.5 ± 9.4 [17, 42]
Body mass (kg)	79.8 ± 23.9 [51.2, 127.6]	66.9 ± 9.6 [48.2, 84.6]	64.8 ± 10.1 [42.6, 82.9]	64.3 ± 11.3 [51.4, 91.0]	66.0 ± 7.7 [56.1, 79.5]
Body height (cm)	171.2 ± 11.3 [162, 180]	169.9 ± 5.8 [148, 182]	168.8 ± 7.8 [156, 192]	172.3 ± 10.7 [156, 192]	171.5 ± 8.2 [164, 183]
BMI (kg/m^2^)	26.9 ± 7.7 [18.0, 43.1]	23.1 ± 2.7 [18.4, 27.9]	22.6 ± 2.4 [19.4, 28.0]	21.5 ± 1.7 [18.6, 24.7]	22.4 ± 1.8 [20.9, 25.0]

BMI: body mass index; ranges are reported as [minimum, maximum].

**Table 2:** Concurrent agreement of heart rate variability devices in the three assessed positions.

		ICC [95% CI]	Lower LoA [95% CI]	Upper LoA [95% CI]	SEM	CV (%)	SDC
Time-domain							
Supine	Mean RR (ms)	1.000 [0.999, 1.000]	−8.087 [−9.886, −6.288]	6.638 [4.839, 8.437]	2.68	0.27	7.43
	RMSSD (ms)	0.999 [0.998, 0.999]	−3.788 [−4.601, −2.975]	2.865 [2.052, 3.678]	1.23	1.93	3.42
	SDNN (ms)	0.997 [0.996, 0.999]	−4.725 [−5.857, −3.593]	4.544 [3.412, 5.677]	1.66	2.91	4.59
Seated	Mean RR (ms)	0.999 [0.999, 1.000]	−10.634 [−13.354, −7.915]	11.626 [8.907, 14.346]	3.99	0.44	11.07
	RMSSD (ms)	0.996 [0.994, 0.998]	−5.597 [−6.959, −4.234]	5.558 [4.196, 6.921]	1.99	3.70	5.52
	SDNN (ms)	0.996 [0.993, 0.998]	−5.176 [−6.565, −3.787]	6.192 [4.804, 7.581]	2.06	3.80	5.72
Standing	Mean RR (ms)	0.999 [0.998, 0.999]	−13.591 [−16.836, −10.346]	12.973 [9.728, 16.218]	4.75	0.57	13.17
	RMSSD (ms)	0.998 [0.996, 0.999]	−3.938 [−4.891, −2.986]	3.862 [2.909, 4.814]	1.39	3.52	3.86
	SDNN (ms)	0.990 [0.983, 0.994]	−7.571 [−9.497, −5.645]	8.195 [6.269, 10.120]	2.82	6.21	7.83
Frequency-domain							
Supine	LF (ms^2^) *	0.999 [0.998, 0.999]	0.791 [0.746, 0.839]	1.281 [1.208, 1.359]	80.62	5.16	223.46
	HF (ms^2^) *	0.998 [0.997, 0.999]	0.788 [0.747, 0.832]	1.222 [1.158, 1.290]	155.88	6.49	432.08
Seated	LF (ms^2^) *	0.995 [0.991, 0.997]	0.717 [0.660, 0.780]	1.420 [1.306, 1.543]	278.50	11.75	771.95
	HF (ms^2^)	0.996 [0.993, 0.998]	−507.0 [−625.1, −388.9]	459.9 [341.8, 578.1]	173.53	11.13	481.01
Standing	LF (ms^2^) *	0.997 [0.994, 0.998]	0.707 [0.658, 0.760]	1.276 [1.187, 1.372]	139.13	8.62	385.65
	HF (ms^2^) *	0.997 [0.995, 0.999]	0.742 [0.683, 0.805]	1.453 [1.338, 1.577]	90.87	8.72	251.88
Non-linear							
Supine	SD1 (ms) *	0.998 [0.997, 0.999]	0.911 [0.893, 0.929]	1.071 [1.050, 1.093]	1.18	2.59	3.28
	SD2 (ms)	0.996 [0.992, 0.998]	−7.027 [−8.789, −5.266]	7.391 [5.629, 9.152]	2.58	3.98	7.15
Seated	SD1 (ms)	0.993 [0.988, 0.996]	−4.983 [−6.303, −3.663]	5.822 [4.502, 7.141]	1.95	5.08	5.41
	SD2 (ms)	0.996 [0.992, 0.998]	−6.895 [−8.760, −5.029]	8.376 [6.511, 10.242]	2.78	4.21	7.70
Standing	SD1 (ms) *	0.997 [0.995, 0.999]	0.905 [0.882, 0.928]	1.114 [1.086, 1.142]	1.08	3.84	3.01
	SD2 (ms)	0.985 [0.974, 0.991]	−11.465 [−14.413, −8.517]	12.668 [9.720, 15.617]	4.33	7.57	12.01

CV: coefficient of variation; HF: high frequency; ICC: intraclass correlation coefficient; LF: low frequency; LoA: limits of agreement; Mean RR: the mean of the time interval between 2 consecutive R waves; RMSSD: square root of the mean of the sum of the squares of successive normal R–R interval differences; SD1: standard deviation of points perpendicular to the line of identity in the Poincaré plot; SD2: standard deviation of points along the line of identity in the Poincaré plot; SDC: smallest detectable change; SDNN: standard deviation of all normal R–R intervals; SEM: standard error of measurement; [95% CI]: 95% confidence interval; * LoA and 95% CIs were calculated on the natural-log scale and back-transformed as accelerometry-to-Polar H10 ratios.

## Data Availability

The data are available upon request to the first author.

## Abbreviations

The following abbreviations are used in this manuscript:

HRV	Heart rate variability
ICC	Intraclass correlation coefficient
SEM	Standard error of measurement
SDC	Smallest detectable change
CV	Coefficient of variation
SD	Standard deviation
Mean RR	The mean of the time interval between two consecutive R waves
RMSSD	Square root of the mean of the sum of the squares of successive normal R-R interval differences
SDNN	Standard deviation of all normal R-R intervals
HF	High frequency
LF	Low frequency
SD1	Standard deviation of points perpendicular to the line of identity in the Poincaré plot
SD2	Standard deviation of points along the line of identity in the Poincaré plot
LoA	Limits of agreement
95% CI	95% confidence interval
